# Identifying SARS-CoV-2 Lineage Mutation Hallmarks and Correlating Them With Clinical Outcomes in Egypt: A Pilot Study

**DOI:** 10.3389/fmolb.2022.817735

**Published:** 2022-03-08

**Authors:** Sara H. A. Agwa, Hesham Elghazaly, Mahmoud Shawky El Meteini, Yahia A. Yahia, Radwa Khaled, Aya M. Abd Elsamee, Reham M. Darwish, Shaimaa M. Elsayed, Hala Hafez, Basma S. Mahmoud, Fouda EM, Marwa Matboli

**Affiliations:** ^1^ Clinical Pathology and Molecular Genomics Unit of Medical Ain Shams Research Institute (MASRI), Faculty of Medicine, Ain Shams University, Cairo, Egypt; ^2^ Oncology Department, Medical Ain Shams Research Institute (MASRI), Cairo, Egypt; ^3^ Department of General Surgery, The School of Medicine, University of Ain Shams, Abbassia, Cairo, Egypt; ^4^ Biochemistry Department, Faculty of Pharmacy, Misr University for Science and Technology, Giza, Egypt; ^5^ Biotechnology/Biomolecular Chemistry Program, Faculty of Science, Cairo University, Cairo, Egypt; ^6^ Biochemistry Department, Faculty of Medicine, Modern University for Technology and Information, Cairo, Egypt; ^7^ Biochemistry and Molecular Genomics Unit of Medical Ain Shams Research Institute (MASRI), Ain Shams University, Cairo, Egypt; ^8^ Clinical Pathology Department, Infection Control Unit, University of Ain Shams, Cairo, Egypt; ^9^ Pediatric Department, Faculty of Medicine, Ain Shams University, Cairo, Egypt; ^10^ Medicinal Biochemistry and Molecular Biology Department, Faculty of Medicine, University of Ain Shams, Cairo, Egypt

**Keywords:** SARS-CoV-2, mutation, NGS, *C36* lineage, Egypt

## Abstract

The SARS-CoV-2 pandemic has led to over 4.9 million deaths as of October 2021. One of the main challenges of creating vaccines, treatment, or diagnostic tools for the virus is its mutations and emerging variants. A couple of variants were declared as more virulent and infectious than others. Some approaches were used as nomenclature for SARS-CoV-2 variants and lineages. One of the most used is the Pangolin nomenclature. In our study, we enrolled 35 confirmed SARS-CoV-2 patients and sequenced the viral RNA in their samples. We also aimed to highlight the hallmark mutations in the most frequent lineage. We identified a seven-mutation signature for the SARS-CoV-2 *C36* lineage, detected in 56 countries and an emerging lineage in Egypt. In addition, we identified one mutation which was highly negatively correlated with the lineage. On the other hand, we found no significant correlation between our clinical outcomes and the *C36* lineage. In conclusion, the *C36* lineage is an emerging SARS-CoV-2 variant that needs more investigation regarding its clinical outcomes compared to other strains. Our study paves the way for easier diagnosis of variants of concern using mutation signatures.

## Introduction

The World Health Organization declared COVID-19 as a pandemic on 11 March 2020 since it appeared as a cluster of pneumonia with unknown cause in Wuhan, Hubei Province, China, in December 2019 (Huang et al., 2020) (Wu et al., 2020). The symptoms range from asymptomatic presentations to dizziness, dry cough, fever, and shortness of breath (Mirzaei et al., 2020) and peak at long-term damage in the lungs (Del Rio et al., 2020) and death in many cases.

The world has face huge economic losses due to lockdown restrictions (Verschuur et al., 2021). Non-pharmaceutical interventions (NPI) against the coronavirus helped to reduce its incidences like mask-wearing, personal hygiene (Cowling et al., 2020), and physical distancing (Huang et al., 2021).

SARS-CoV-2 is a positive-sense RNA virus in the order *Nidovirales*, family Coronaviridae with an approximately 30 kb single-stranded RNA genome (Elena and Sanjuán, 2005) RNA viruses possess a high mutation rate that is higher than their hosts which impacts viral pathogenicity, infectivity, and transmissibility. The SARS-CoV-2 RNA genome encodes 16 non-structural proteins (*NSP*) and at least 10 structural proteins including spike (*S*), *ORF3a*, envelop (*E*), membrane (*M*), open reading frame 6 (*ORF6*), *ORF7a*, *ORF7b*, *ORF8*, nucleocapsid (*N*), and *ORF10* (Cagliani et al., 2020; Yuan et al., 2021).

The severe morbidity and mortality worldwide worried medical and scientific societies and forced them to make intense and rapid strategies for vaccine development (Zhou et al., 2020). After the isolation and sequencing of the SARS-CoV-2 genome, different genetic clades appeared in Hong Kong in the first 2 months after the identification of SARS-CoV-2 including the *V*, *S*, and *L* clades (To et al., 2021), these variants were thought to worsen vaccine potency (Mahase, 2021) and also cause reinfections (Zucman et al., 2021).

Baud et al. supported the hypothesis that the mortality of SARS-CoV-2 changes depending on geographical regions as they reported that the death rate incidence outside of China is three times higher compared to death rates in China (Baud et al., 2020), The different policies in each country influence the infection rates, and herd immunity of different genetic populations is also considered an important factor.

The persistence of COVID-19 accumulates mutations that paralyze the drug development process albeit with the massive efforts of pandemic trapping. Many studies reported specific mutations related to geographical regions: *Val483Ala* and *Gly476Ser* are primarily observed in samples from the United States, whereas *Val367Phe* is found in samples from China, Hong Kong Special Administrative Region, France, and the Netherlands (Ou et al., 2021).

Varying patients’ responses to different variants of SARS-CoV-2 revealed the need to trace the different variants of SARS-CoV-2 and to study their transmissibility and virulence. For instance, some variants were found to be more virulent and transmissible such as Alpha, Delta, Gamma, Kappa, and Omicron (Christie, 2021; Otto et al., 2021).

Identifying mutations and correlating between them help to identify key features of different strains. Correlating significant mutations and relating them to clinical findings aid in highlighting variants of concern that exhibit more virulence and resistance.

Next-generation sequencing (NGS) techniques are the milestone that can easily identify new and virulent mutations which may help in solving the massively widespread and rapid mutation rates of the pandemic. In addition, NGS may help in tracing the mutation rates and the evolutionary clock of the virus. NGS tools also provide lower cost and unbiased methods for detecting pathogens, with high-speed sequencing that can sequence billions of nucleic acid fragments at once and aid in vaccine and antiviral research, phylogenetic analysis, viral transmission tracing, and pathogen evolution monitoring (Udugama et al., 2020; John et al., 2021).

In this study, we aimed to correlate mutations with lineages to identify the hallmarks of identified lineages. This identification may lead to spotlighting the variants of concern. This method of identification may lead to better treatments, vaccine development, better viral diagnostic approaches, risk categorization, and predict the possible future mutation mechanisms in Egypt. In addition, we aimed to highlight the virulence of viral lineages in Egypt by correlating them with our clinical outcomes. This correlation may lead to a better prognosis of specific viral lineages that may help in clinical decisions and reduce the economic burden nationally and internationally.

## Materials and Methods

### Ethics Statement

The study protocol was approved by the Ethical Committee of Ain Shams University, approval number: (FMASU P17a/2020). Samples used in this study were previously ethically approved with informed patients’ consent in an ongoing project. Reports from hospital records were also used.

### Clinical Sample Collection and Processing

Between April 2020 and August 2020, nasopharyngeal (NP) and oropharyngeal swabs were gathered from 35 patients positive for SARS-CoV-2. Inclusion criteria included patients with symptoms and those confirmed to be SARS-CoV-2-positive by real-time PCR; weight ≥10 kg; and age ≥3 years old. Based on the fact that all populations are susceptible to SARS-CoV-2 infection, only individuals or family members who did not give consent to participate were excluded. Also, non-Egyptian patients were excluded. Patients inside every group were sub-grouped according to the severity of symptoms: Mild, moderate, and severe based on their criteria for patient selection including age, sex, and the severity of the disease according to the COVID-19 Treatment Guidelines Panel, National Institutes of Health (COVID-19 Treatment Guidelines Panel, 2019). Fever, cough, and weariness are common symptoms of mild infections. Moderate individuals may suffer breathing difficulties or mild pneumonia. Severe cases may experience severe pneumonia, organ failure, and possible death (World Health Organization, 2021).

Oropharyngeal and nasopharyngeal swab samples were collected from hospitalized patients from different places in Egypt (Medany Hospital, Demerdash Hospital, Central Labs, Qalyobeyyah, and Internal Medicine Hospital) as set out in the guidelines of the Ministry of Health and Population in Egypt. Patients had completed a questionnaire that covered age, history of fever and/or respiratory symptoms, traveling history, any underlying lung disease, history of chronic or immune-compromised conditions, and outcome. The records were used retrospectively to assess the patients’ clinical characteristics and severity to categorize their cases into (mild, moderate, or severe).

Samples placed in a centrifuge tube were labeled with the patient unique ID and containing 2 ml of viral transport media (VTM) were agitated vigorously for 10 s using a vortex mixer. VTM was split into two pre-labeled, sterile cryovials with the correct patient ID. One cryovial was immediately placed in a freezer (−80°C), while the other cryovial was used for molecular studies at Medical Ain Shams Research Institute (MASRI) Molecular Genomic Labs.

### Viral RNA Extraction and SARS-Cov-2 Detection by QRT-PCR

Viral RNA isolation was performed using a MagMax viral/pathogen nucleic acid isolation kit (ThermoFisher Scientific, Waltham, MA, United States). Real-time reverse transcription-polymerase chain reaction (RT-PCR) was used for simultaneous amplification of four target genes, including nucleocapsid protein (*N*), and open reading frame 1ab (*ORF1ab*), *ORF3a*, and *S* proteins. COVID-19 detection was done using ProLab/CerTest Biotech ViaSure SARS-CoV-2. The Real-time PCR detection Kit (VS-NCO296T, CerTest Biotec, S.L, Spain, Catalogue number VS-NCO213L) was used in an Applied Biosystems™ 7500 Fast Real-Time PCR System following the cycling and fluorescence acquisition parameters detailed in the manufacturer’s protocol. Five microliters of RNA was isolated from clinical samples and checked for quantity, purity, and quality by a Qubit^®^ 2.0 Fluorometer (Qubit^®^ RNA Assay Kit, Life Technologies, CA, United States) High Sensitivity Kit (Invitrogen, Carlsbad, CA, United States). The RNA was then used in each real-time PCR reaction, with a final volume of 20 µl. Samples were processed with appropriate negative, internal, and positive controls. Samples were run in duplicate. Real-Time Detection Systems analysis was done by Applied biosystem 7500 Real-Time PCR Software v2.0. The cycle threshold value of [C t] below 34 was considered to be positive. Compliance with the WHO-recommended research protocol confirmatory laboratory testing was carried out.

### Viral Genome Sequencing for Positive SARS-CoV-2 Samples by Targeted NGS

After viral RNA isolation, reverse transcription and cDNA synthesis were completed. After RNA extraction and assessment, RNA was reverse-transcribed using the SuperScript™ VILO™ cDNA Synthesis Kit (Cat. No.11754050; Invitrogen, Grand Island, United States), according to the product protocol. Targets for sequencing were obtained based on the Ion AmpliSeqTM SARS-CoV-2 Panel (ThermoFisher, Waltham, MA, United States). Library preparation was made using the Ion AmpliSeqTM Library Kit Plus (ThermoFisher, Waltham, MA, United States) (Cat. Nos. 4488990). Primer pool 1 and two target amplification reactions were combined and amplicons were partially digested; barcode adapters were ligated and purified using the Ion Xpress™ Barcode Adapters 1–96 Kit (Cat. No. 447451), then libraries were quantified using the Ion Library TaqMan™ Quantitation Kit (Cat. No. 4468802), the Ion 530™ Kit–Chef (Cat. No. A34461), according to the user guide.

The libraries were sequenced on the Ion GeneStudio S5 Series System platform with an Ion AmpliSeq SARS-CoV-2 Research Panel (ThermoFisher Scientific, Waltham, MA, United States) that contains two pools with amplicons ranging from 125 bp to 275 bp in length and includes >99% of the SARS-CoV-2 genome, covering all serotypes. A complete genome (29,903 nucleotides) was assembled, with 0.13% unique mutations to the other viral genomes.

### Bioinformatics Analysis

Using BLAST against the NCBI *betacoronavirus* database, the closest matches were several sequences with a bit score of 33,479, including, for example, isolate SARS-CoV-2/human/USA/VA-DCLS-0556/2020 (99.9%), accession (*MT739463*). The assembled genome along with the other SARS-CoV-2 genomes obtained and clustered from GISAID was aligned using MAFFT (Katoh et al., 2002).

We used Torrent Suite Software–provided with the Ion AmpliSeq SARS-CoV-2 research panel–for generating *de novo* full-length sequences from raw samples’ sequences. Sequence genes’ annotations were carried out using the COVID19AnnotateSnpEff plugin as instructed by the provider’s manual.

Phylogenetic analysis was done on all 35 sequences using the MAFFT (version 7) command-line tool (Katoh et al., 2002). The unweighted pair group method with arithmetic mean (UPGMA) was used for constructing the phylogenetic tree, and the iTOL (version 5) online tool was used to visualize it (Letunic and Bork, 2021).

### Correlation Analysis Between Mutations

The analysis was made using R (version 3.6.2). Missense mutations were plotted as a matrix against samples. If a mutation is present in a sample, it was given a value of 1. If the sample matched the reference at a site of mutation, it was given a zero value. Spearman’s correlation coefficients were computed for network analysis using the qgraph R package (version 1.6.9) (Epskamp et al., 2012).

### Clustering Analysis and Grouping Samples

Samples were divided into two clusters based on the Euclidean distance between samples. Clustering was plotted using “heatmap.2” under the “gplots” R package (version 2.17.0). Sample grouping was carried out based on the clusters formed into two groups, A and B, based on the genetic variations.

### Correlation Analysis Between Mutation Clusters and Clinical Outcomes

Correlation analyses were made between clinical outcomes and the two clusters. *Shapiro-Wilk*’s test was used for normality and *F*-test for homogeneity for every outcome. The most appropriate test was used for every outcome according to the previous assumptions.

### Samples Classification and Correlated Mutations Effects

We used the Phylogenetic Assignment of Named Global Outbreak Lineages (Pangolin) (version 3.1.5) command-line tool to classify our samples (Rambaut et al., 2020). We used the Sorting Intolerant from Tolerant (SIFT) web server (version 6.2.1) to predict the effect of correlated mutations on the protein function (Sim et al., 2012).

## Results

A total of 35 samples were selected based on quality checks comprising 15 men and 20 women during the early months of the pandemic (Table 1).

**TABLE 1:** Group classifications according to gender, severity, and age with clinical outcomes of patients.

	Group A *N* = 16			Group B *N* = 19			Test of significance
Sex							
Male: *N* = 15 (43%)	7 (44%)			8 (42%)			*X* ^*2*^ = 0.0667
Female: *N* = 20 (57%)	9 (56%)			11 (58%)			*P* = 0.7963
Severity							
Mild	2			1			*W* = 194
Moderate	5			2			*P* = 0.0827
Severe	9			16			
Comorbidities						
Diabetes mellitus (DM)	7			5			*X* ^*2*^ = 0.33
							*P* = 0.56
Hypertension (HTN)	7			5			*X* ^*2*^ = 0.33
							*P* = 0.56
DM + HTN	4			3			*X* ^*2*^ = 0
							*P* = 1
Bronchial asthma	2			2			*X* ^*2*^ = 0
							*P* = 1
	Mean	±SD	Standard error	Mean	±SD	Standard error	Test of Sig.
Age/years	35.73	27.61	7.13	18.68	22.67	5.20	*W* = 85
							*P* = 0.0476
TLC (thousands/cmm^3^)	9.93	4.39	1.098	14.14	18.53	4.25	*W* = 152
							*P* = 1
Hemoglobin (g/dl)	10.23	1.76	0.44	10.45	2.32	0.534	*W* = 130
							*P* = 0.47
Platelets (thousands/cmm)	249.06	133.21	30.56	253.36	85.66	21.41	*T* test = 0.1153
							0.9089
Ferritin (mg/L)	394.71	245.47	61.36	334.07	401.67	92.15	W = 110.5
							P = 0.1744
Lactate dehydrogenase (LDH) (U/L)	412.71	188.47	47.11	395.68	189.68	43.51	*t* test = -0.266
							P = 0.7923
D-dimer(mg/L)	1.40	1.40	0.35	2.84	5.54	1.27	W = 131
							P = 0.497

Total leukocyte count (TLC) (thousands/cmm^3^), hemoglobin (g/dl), platelets (thousands/cmm^3^), ferritin (mg/L), and lactate dehydrogenase (LDH) (U/L). *X* ^*2*^: *Chi-square* test, *W*: Mann–Whitney *U* test, *P*: *p*-value, and *T-test*: Student *t*-test.

Patients’ severity of symptoms was termed mild, moderate, or severe (Table 1) based on their age, sex, and the severity of the disease.

In total, 160 modifications were recorded and distributed across four genomic regions; *ORF1ab* comprises the longest SARS-CoV-2 gene (approximately 24 kb), corresponding to a polyprotein made up of 16 non-structural proteins (*NSP1-16*), we found that over 56% of all mutations were recorded in this *ORF1ab* specifically in positions 2,841, 10,097, 11,083, 17,766, 4,002, 12,534, and 13,536, this was followed by the spike (*S*) protein in positions 23,403 and 23,593 and nucleocapsid (*N*) protein in positions 28,881 and 28,908 with the lowest number of variants found in *ORF3a* coding genes in position 25,563 as represented in Table 2. Moreover, c.2576C > T (p. *Asp*614*Gly*) in S was the most abundant missense mutation among samples, found in 29 samples (Table 2).

**TABLE 2:** Frequent nucleotide and amino acid modifications in analyzed genomes.

Gene change	Position	Gene	Protein change	Counts
*c.2576C > T*	2,841	*ORF1ab*	*p.Ala859Val*	4
*c.608_610delGGGinsAAC*	28,881	*N*	*p.ArgGly203LysArg*	15
*c.1841A > G*	23,403	*S*	*p.Asp614Gly*	29
*c.171G > T*	25,563	*ORF3a*	*p.Gln57His*	14
*c.2031G > T*	23,593	*S*	*p.Gln677His*	14
*c.635G > T*	28,908	*N*	*p.Gly212Val*	18
*c.9832G > A*	10,097	*ORF1ab*	*p.Gly3278Ser*	16
*c.10818G > T*	11,083	*ORF1ab*	*p.Leu3606Phe*	4
*c.17501C > T*	17,766	*ORF1ab*	*p.Ser5834Phe*	4
*c.3737C > T*	4,002	*ORF1ab*	*p.Thr1246Ile*	14
*c.12269C > T*	12,534	*ORF1ab*	*p.Thr4090Ile*	13
*c.13271C > T*	13,536	*ORF1ab*	*p.Thr4424Ile*	15

**FIGURE 1:**
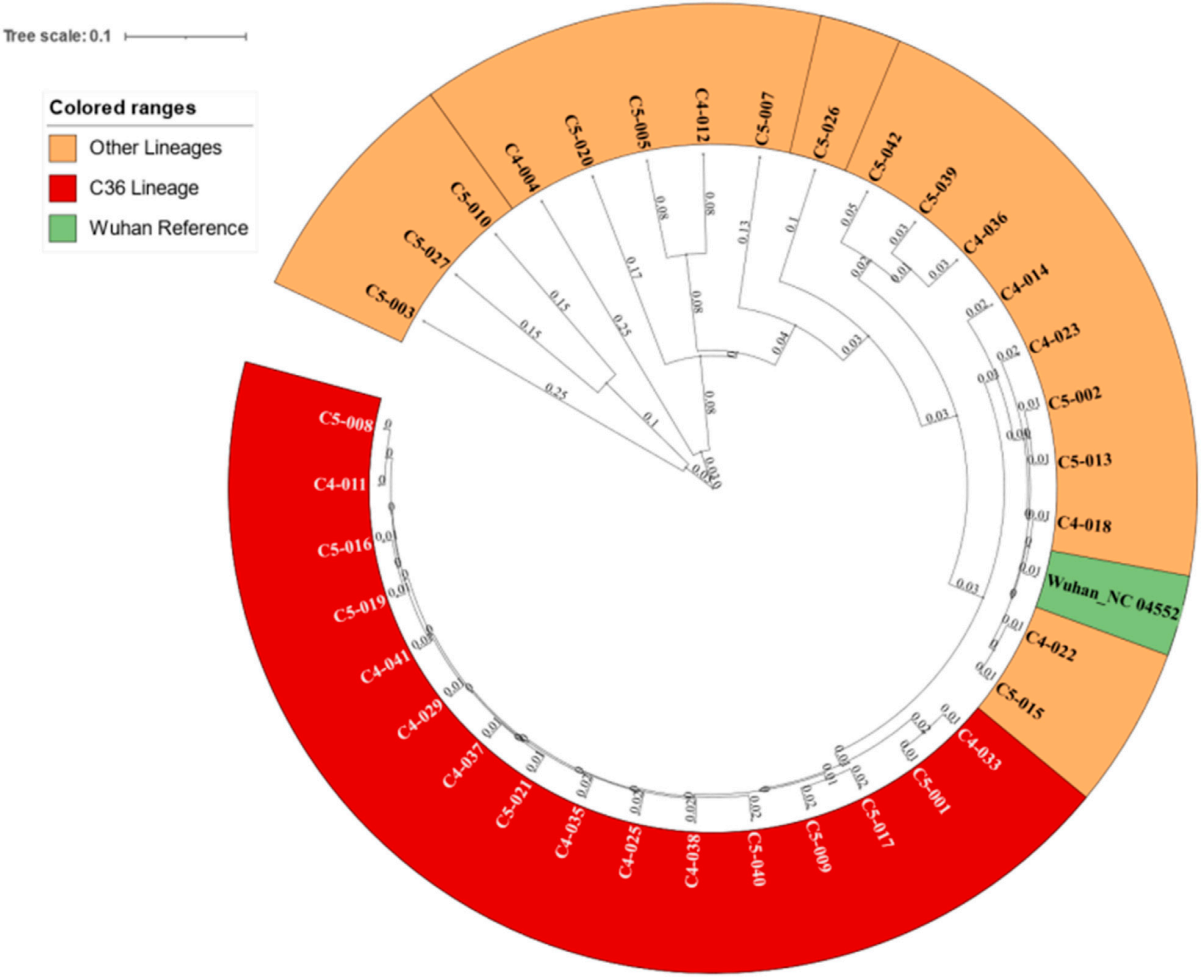
Phylogenetic tree for the 35 samples revealing the *C36* clade and its distance from other lineages.

Phylogenetic analysis revealed the distinction of the *C36* lineage from other lineages forming a clade of 16 leaves (Figure 1).

### Correlation Analysis Between Mutations

**FIGURE 2:**
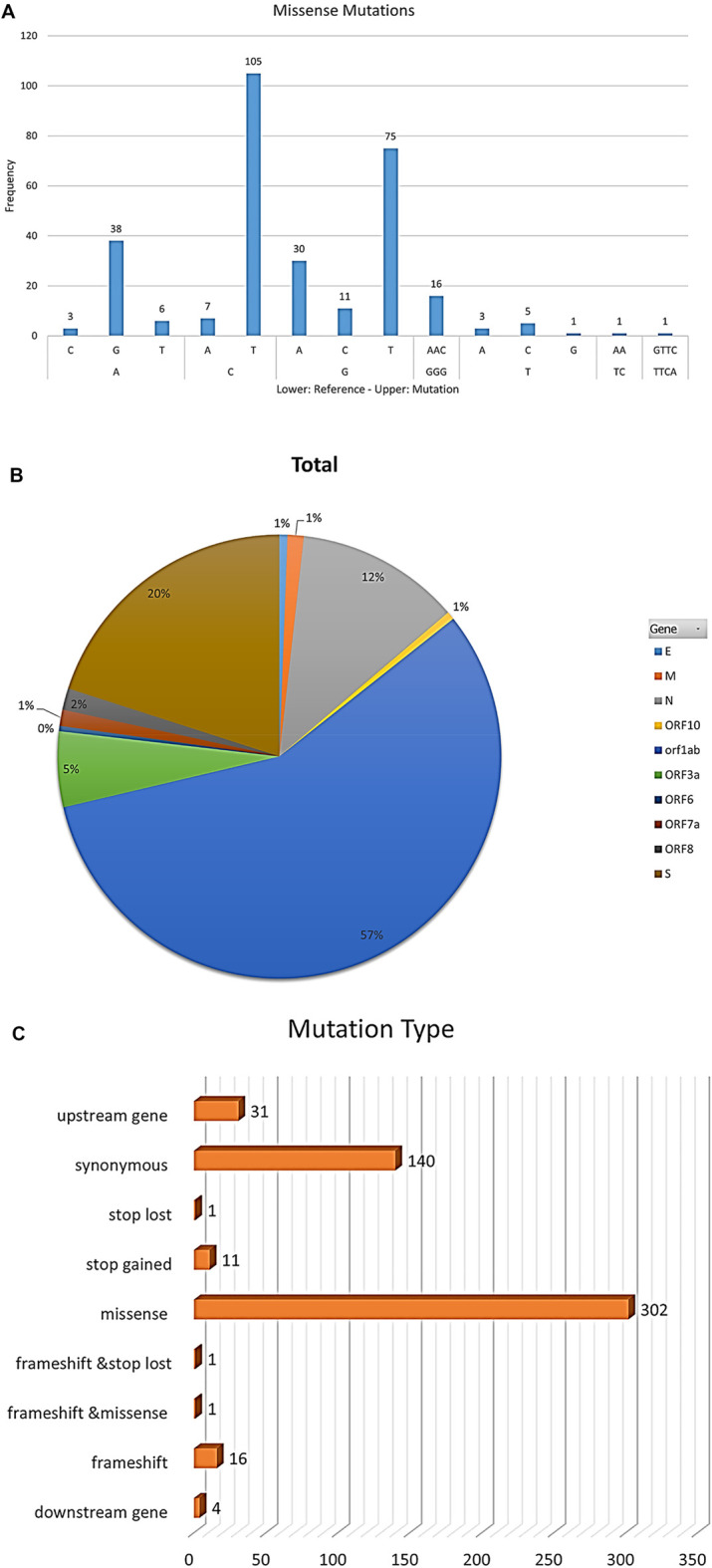
The figure represents statistics of mutation frequencies in all samples. **(A)** Bar plot represents frequencies of nucleotide mutations where the x-axis lower row represents reference nucleotide while the x-axis upper row represents the mutated nucleotide in samples. Frequency is represented on the y-axis. **(B)** Pie-chart represents mutations’ total frequencies in genes in all samples. **(C)** Bar plot represents mutations’ total frequencies per mutation type.

The most frequent mutations were from cytosine or guanine to thymidine in all samples (Figure 2A) that represented more than 56% of mutations in all samples with a frequency of 302 mutations (Figure 2C). About 56% of mutations appeared in *ORF1ab* (Figure 2B).

### Clusters Analysis and Grouping Samples

**FIGURE 3:**
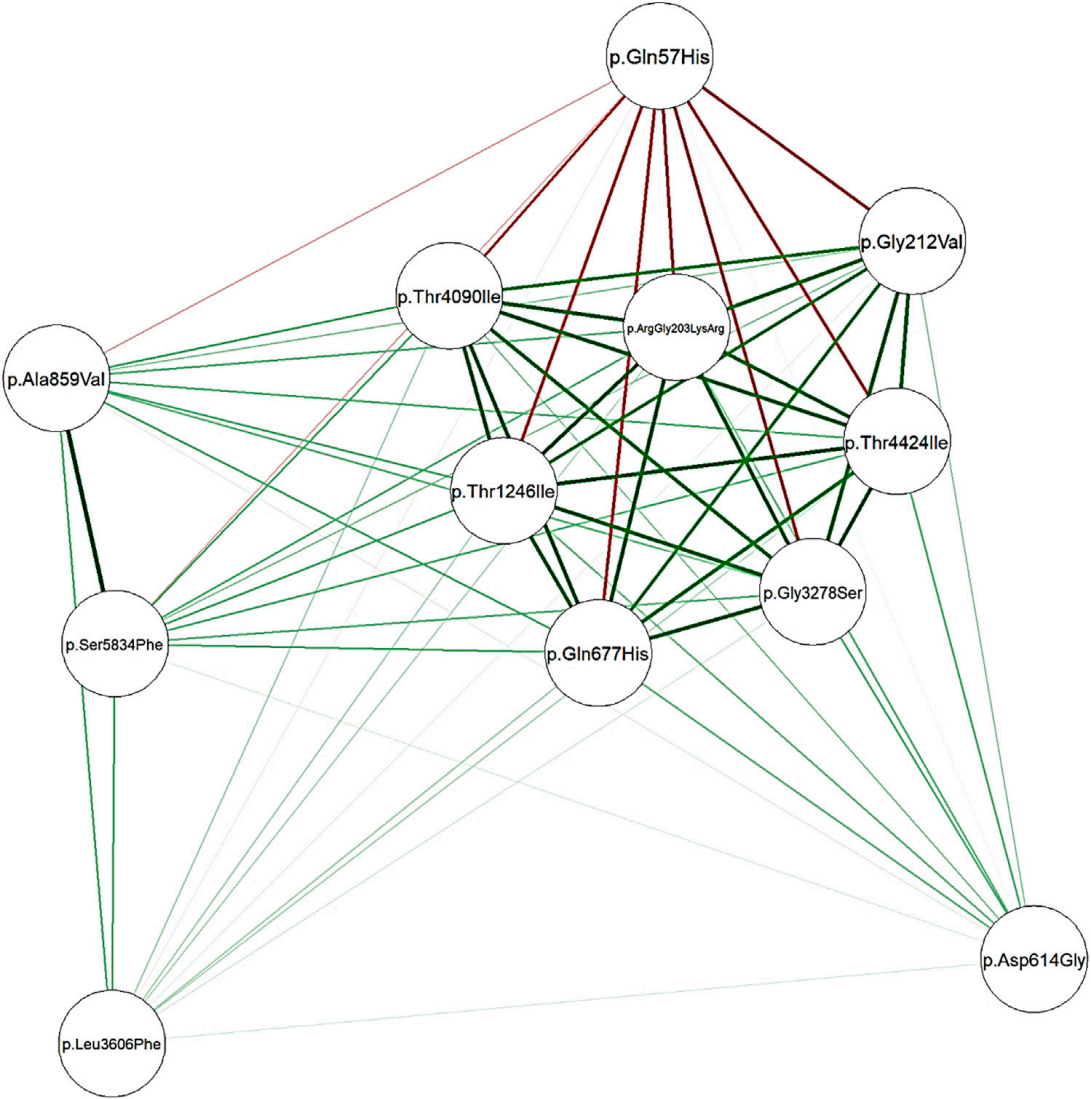
Network plotted based on Spearman’s correlation matrix between mutations. Green edges represent a positive correlation coefficient while red edges represent a negative correlation. Intense color represents a higher correlation while the color fades when correlation falls to zero.

**FIGURE 4:**
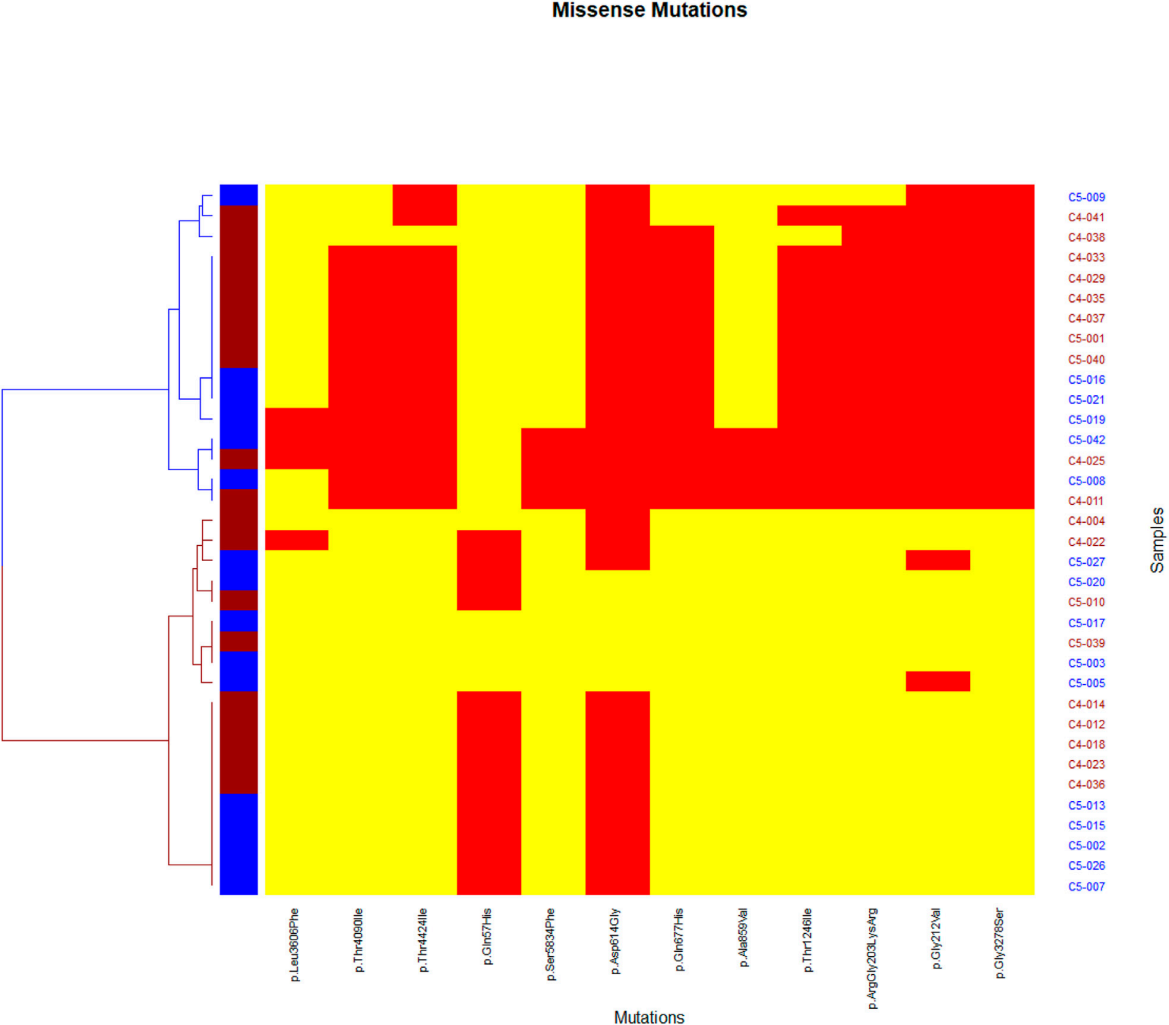
Heatmap representing missense mutations on the *x*-axis and samples on the *y*-axis. A yellow color indicates the absence of the mutation in the sample while a red color indicates the presence of the mutation. Two clades appear, a blue clade which we considered as a group **(A)**, and a red clade as group **(B)**.

Network analysis showed a high positive correlation between seven mutations in *Nucleoprotein*, *spike*, and *ORF1ab* genes, and a high negative correlation between the seven mutations and one mutation in the *ORF3a* gene (Figure 3). The dendrogram (Figure 4) showed two clades of samples; a clade that carried the 7 correlated mutations was composed of 16 samples (group A); the second clade was composed of 19 samples carrying the negatively correlated mutation (*Gln*57*His*) (group B).

### Correlation Analysis Between Patient Groups and Clinical Outcomes

**FIGURE 5:**
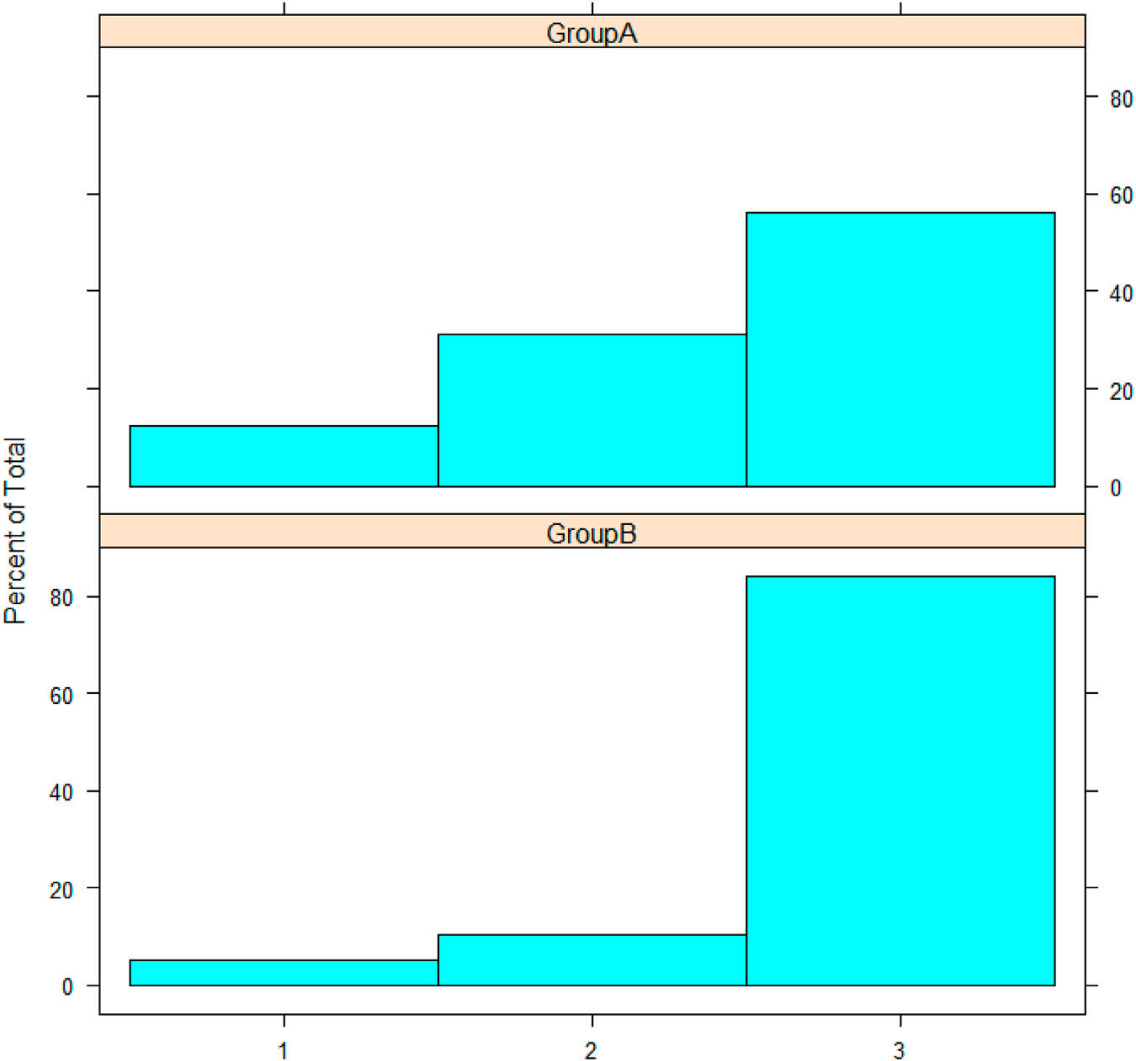
The histogram represents severity; the *y*-axis represents the frequency percentage in each group; the *x*-axis represents severity as numbers: 1, 2, and 3 for mild, moderate, and severe, respectively.

Patients presented with comorbidities such as diabetes mellitus, hypertension, or both were reported. Previously diagnosed asthmatic patients were reported as having a comorbidity as well. Cough was reported in all samples, analyzed using Mann-Whitney’s *U* test, and no statistically significant difference was observed between the two groups (*p*-value = 0.4783). The severity of symptoms was reported in all samples (Figure 5), and Mann-Whitney’s *U* test was used. The two groups showed no statistical significance in the severity outcome (*W* = 194, *p*-value = 0.08277), Laboratory outcomes were reported such as (TLC, hemoglobin, platelets, ferritin, lactate dehydrogenase, D-dimer); statistical tests were chosen after testing assumptions such as normality (using Shapiro-Wilk’s test) and homogeneity of variance (using F-test). Based on the prior assumptions, Mann-Whitney U, Student t, and Chi-square tests were used as in Table 1. No statistical significance was found between group A and group B (Table 1).

### Samples Classification and Correlated Mutations Effects

Phylogenetic analysis revealed 16 sequences under the same clade that were identified as *C36* lineages using further analysis (Figure 1).

Group A samples were all classified as lineage *C36* according to Pangolin. Group B samples were classified under *A* and *B* lineages and their sub-lineages. In group B, the *Gln57His* mutation at *ORF3a* was predicted to affect the function of the protein with a high score (0.00). In group A, the *Gly204Arg* mutation in the nucleocapsid protein and *Thr1246Ile* and *Thr4090Ile* mutations in *ORF1ab* were predicted to affect their proteins with scores of 0.02, 0.00, and 0.00, respectively. However, other correlated mutations on protein function were tolerated according to the SIFT algorithm.

### Data Availability Statement

All sequenced data were submitted into the SARS-CoV-2 Global Initiative on Sharing All Influenza Data (GISAID) database as shown in Table 3. In all figures, we used the corresponding abbreviations (Table 3) throughout the study.

**TABLE 3:** SARS-CoV-2 Egyptian patients’ samples sequenced and deposited with GISAID.

Strain	GISAID acc. No.	Abbreviations
hCoV-19/Egypt/MASRI-C4-038/2020	EPI_ISL_1165081	C4-038
hCoV-19/Egypt/MASRI-C4-011/2020	EPI_ISL_1141525	C4-011
hCoV-19/Egypt/MASRI-C4-012/2020	EPI_ISL_1165085	C4-012
hCoV-19/Egypt/MASRI-C4-041/2020	EPI_ISL_1109486	C4-041
hCoV-19/Egypt/MASRI-C4-014/2020	EPI_ISL_1165086	C4-014
hCoV-19/Egypt/MASRI-C4-018/2020	EPI_ISL_1165087	C4-018
hCoV-19/Egypt/MASRI-C4-022/2020	EPI_ISL_1165082	C4-022
hCoV-19/Egypt/MASRI-C4-023/2020	EPI_ISL_1165083	C4-023
hCoV-19/Egypt/MASRI-C4-025/2020	EPI_ISL_1098839	C4-025
hCoV-19/Egypt/MASRI-C4-029/2020	EPI_ISL_1165078	C4-029
hCoV-19/Egypt/MASRI-C4-004/2020	EPI_ISL_1165084	C4-004
hCoV-19/Egypt/MASRI-C4-033/2020	EPI_ISL_1165079	C4-033
hCoV-19/Egypt/MASRI-C4-035/2020	EPI_ISL_1109484	C4-035
hCoV-19/Egypt/MASRI-C4-036/2020	EPI_ISL_1109485	C4-036
hCoV-19/Egypt/MASRI-C4-037/2020	EPI_ISL_1165080	C4-037
hCoV-19/Egypt/MASRI-C5-001/2020	EPI_ISL_1109624	C5-001
hCoV-19/Egypt/MASRI-C5-010/2020	EPI_ISL_1167190	C5-010
hCoV-19/Egypt/MASRI-C5-039/2020	EPI_ISL_1167196	C5-039
hCoV-19/Egypt/MASRI-C5-040/2020	EPI_ISL_1586895	C5-040
hCoV-19/Egypt/MASRI-C5-013/2020	EPI_ISL_1109628	C5-013
hCoV-19/Egypt/MASRI-C5-042/2020	EPI_ISL_1167197	C5-042
hCoV-19/Egypt/MASRI-C5-015/2020	EPI_ISL_1109627	C5-015
hCoV-19/Egypt/MASRI-C5-016/2020	EPI_ISL_1109625	C5-016
hCoV-19/Egypt/MASRI-C5-017/2020	EPI_ISL_1167191	C5-017
hCoV-19/Egypt/MASRI-C5-019/2020	EPI_ISL_1109630	C5-019
hCoV-19/Egypt/MASRI-C5-002/2020	EPI_ISL_1109629	C5-002
hCoV-19/Egypt/MASRI-C5-020/2020	EPI_ISL_1167192	C5-020
hCoV-19/Egypt/MASRI-C5-021/2020	EPI_ISL_1167193	C5-021
hCoV-19/Egypt/MASRI-C5-026/2020	EPI_ISL_1167194	C5-026
hCoV-19/Egypt/MASRI-C5-027/2020	EPI_ISL_1167195	C5-027
hCoV-19/Egypt/MASRI-C5-003/2020	EPI_ISL_1167186	C5-003
hCoV-19/Egypt/MASRI-C5-005/2020	EPI_ISL_1167187	C5-005
hCoV-19/Egypt/MASRI-C5-007/2020	EPI_ISL_1167188	C5-007
hCoV-19/Egypt/MASRI-C5-008/2020	EPI_ISL_1109626	C5-008
hCoV-19/Egypt/MASRI-C5-009/2020	EPI_ISL_1167189	C5-009

## Discussion

Sequencing using NGS techniques revealed the blurry areas in the SARS-CoV-2 genome that helped us to make panoramic insights about mutation patterns and explain the mounting infectivity of the virus all over the world. Moreover, these techniques helped us to put forward the right explanation of population re-infection and antigenic consequences (Li et al., 2005).

We analyzed the genomic variants of 35 Egyptian patients during the first wave of the pandemic and divided them into two groups after phylogenetic analysis. The first group (B) included all lineages except *C36* lineage. While group (A) included only sequences that were classified as the *C36* lineage. According to Pangolin, the *C36* lineage first appeared in the United States on 13 March, 2020. However, the highest incidence according to the GISAID database is in the Egyptian population. The *C36* lineage has been detected in at least 56 countries worldwide (Anderson et al., 2021).

The *C36* lineage compromises 34% of all sequenced variants in Egypt, 11% of sequenced variants in Germany, 10% of sequenced variants in the United Kingdom, 7% of sequenced variants in the United States, and 6% of sequenced variants in Denmark until January 2022 according to Pangolin.

Roshdy et al. confirmed the presence of the *C36* lineage early in the pandemic and its evolution into several sub-lineages, including *C.36.1*, *C.36.3*, and *C.36.3.1*, circulating across the Egyptian patients’ genome. They also discovered that mutations in this lineage show potential fitness and pathogenicity in the same manner that mutations in Alpha, Beta, Gamma, Delta, and Omicron (variants of concern) do (Roshdy et al., 2022). The spike mutation related to *C36* lineage *Gln*677*His* in position 23,593 which emerged firstly in the United States confers an advantage in spreading and transmissibility through its position in the S1/S2 boundary upstream furin cleavage site (Hodcroft et al., 2021).

Among the 35 genomes, more than 56% of mutations were missense mutations with a frequency of 302 mutations followed by synonymous mutations with a frequency of 140 mutations and frameshifts with a frequency of 16 mutations (Figure 2C). C > T transitions may be interfered with by cytosine deaminases (Lyons and Lauring, 2017). G > T transversions are more likely to be introduced by oxo-guanine from reactive oxygen species (Li et al., 2006).

Approximately 56% of mutations appeared in *ORF1ab*, which represents more than two-thirds of the genome, controls viral replication, and consequently, these mutations might affect the replication speed of the virus (Yin, 2020).

The most common variant located in the *ORF1ab* region was the missense mutation *c.9832G > A* in region 10,097 that changed glycine amino acid into serine *p.Gly3278Ser* in 16 of our samples. In group (B), *Thr1246Ile* and *Thr4090Ile* mutations in *ORF1ab* were predicted to affect their proteins with scores of 0.00 and 0.00, respectively, and were considered influential parameters that could be possibly linked to the virus’s speed replication and infectivity that contribute to patient severity status.

The *S* protein of SARS-CoV-1 and SARS-CoV-2 forms homo-trimers protruding in the viral surface that facilitates viral entry into the host cells via interacting with angiotensin-converting enzyme 2 (ACE2) which is their main receptor expressed in lower respiratory tract cells (Letko et al., 2020) (Bakhshandeh et al., 2021).

Variants in the spike protein domain showed strong evidence of reducing the neutralization sensitivity to convalescent sera and monoclonal antibodies. These variants potentially lessened the protection afforded by the current vaccines that target the spike region. *Asn439Lys* emerged in Scotland in the spike region and was found to enhance the binding affinity for the ACE2 receptor and reduce the neutralizing activity of some monoclonal antibodies (Thomson et al., 2021) (Greaney et al., 2021) (Wibmer et al., 2021) (Gaebler et al., 2021) (Collier et al., 2021).

We reported that the most frequent modified nucleotides were recorded at position 23,403 in the spike protein *c.1841A > G,* this missense mutation changed aspartic acid into glycine *p.Asp614Gly* found in 29 samples (Table 2) (Alouane et al., 2020) (Lobiuc et al., 2021). The *p.Asp614Gly* mutation firstly appeared in late January in China and rapidly emerged in the global population within a mere 3 months, studies illustrated that the *p.Asp614Gly* mutation confers a moderate advantage for virus transmissibility, infectivity, replication, and elevated fitness; it may explain the high frequency of infections in the Egyptian population (Hou et al., 2020) (Yurkovetskiy et al., 2020).

Cong et al. studied the *N* protein and its impact on the coronaviral life cycle by the contribution to helical ribonucleoproteins formation during RNA genome packaging, modulating viral RNA synthesis during replication and transcription, and modifying metabolism in infected people (Cong et al., 2020). Studies showed that N genes are more conserved and stable, with 90% amino acid homology and fewer mutation frequencies throughout time (Dutta et al., 2020). Changes in the *N* protein charge resulted in enhanced virus replication and ultimately increased infectivity and fitness (Wu et al., 2021). The missense mutation in nucleocapsid phosphoprotein (*N*) in position 28,881 p. *ArgGly*203*LysArg* found in 15 of our patients is already observed in 1,573 samples out of 10,022 SARS-CoV-2 genomes studied from the US, United Kingdom, and Australia (Koyama et al., 2020). The statistical analysis found that the *Gly*204*Arg* mutation in nucleocapsid protein which was found in group B in position 28,881 appeared to influence protein with a score of 0.02. Studies showed that *Arg*203*Lys* and *Gly*204*Arg* are concomitant mutations in the N protein, which are quickly rising in frequency and may be linked to the virus’s infectivity (Zhu et al., 2021). These mutations are found commonly in lineages B.1.1.7 (Alpha) (Caserta et al., 2021; Wu et al., 2021) and P.1 (Gamma) (Faria et al., 2021; Wu et al., 2021). Another mutation p. *Gly*212*Val* in position 28,908 was also found in *N* protein and repeated 18 times.

*ORF3a,* although it is considered an accessory protein, has a vital role in cell surface localization and allows viral entry within the host and possesses immunogenic properties (Zhong et al., 2003) (Liu et al., 2014). Moreover, *ORF3a* is involved in ion channel formation and modulates the release of the virus from the host cell (Liu et al., 2014). Majumdar et al. extensively studied the emerged mutations that appeared in the *ORF3a* protein *in silico* and related these mutations with high mortality rates for SARS-CoV-2 infection through host immune evasion and extreme cytokine storm through JAK-STAT, chemokine, and cytokine-related pathways (Majumdar and Niyogi, 2020).

Interestingly, our data revealed that the *Gln57His* mutation at *ORF3a* affected the function of the protein with a high score (0.00) in group B. Our findings are supported by a study that reports that *ORF3a* mutation *Gln57His* leads to a major truncation of the *ORF3b* protein (Chu et al., 2021).

Zekri et al. previously identified 204 distinct mutations of the Egyptian strains classified under clade B lineage and its sub-lineages, distributed on *ORF1ab*, *S*, *N*, *ORF3a*, *ORF7a*, *ORF8*, *M*, *E*, and *ORF6*. In addition, they found that *Asp*614*Gly* was the most frequent mutation appearing in all their samples. Interestingly *Asp*614*Gly* also appeared in 83% of our samples (Zekri et al., 2021).

Our data showed no statistical significance in the severity outcome between the studied groups (*p*-value = 0.08277).

The laboratory tests investigated in this study included LDH, PLT, Hb, D-dimer, serum ferritin, and platelet counts. Other studies reported the influence of SARS-CoV-2 on those parameters. For instance, LDH was reported to increase in severely symptomatic patients to reach 6-fold its normal values (Henry et al., 2020). Serum ferritin and D-dimer were significantly increased in COVID patients and elevated in more virulent cases (Cheng et al., 2020; Hussein et al., 2021). Platelets and total leucocytes declined in COVID patients as reported by Wool and Miller (Wool and Miller, 2021). However, our study reported no significant correlation between the *C36* mutation signature and clinical outcomes.

## Conclusion

Our study highlights the mutation signature for the *C36* lineage over other lineages. The mutation signature proposes seven positively correlated mutations and one negatively correlated mutation. On the other hand, our study reported no significantly correlated clinical outcomes or predisposing comorbidities that hallmark the *C36* lineage. Interestingly, *C36* tends to affect older patients. However, our clinical findings need more investigation using a larger sample size.

## Institutional Review Board Statement

The study was done based on the guidelines of the Declaration of Helsinki, and received approval from the Research Ethics Committee, Faculty of Medicine, Ain Shams University, Egypt, dated 13/5/2020, FWA 000016584.

## Data Availability

The datasets presented in this study can be found in online repositories. The names of the repository/repositories and accession number(s) can be found in the article/Supplementary Material.
